# Supplementary material for: CytoPipeline and CytoPipelineGUI: a Bioconductor R package suite for building and visualizing automated pre-processing pipelines for flow cytometry data
Source: BMC Bioinformatics. 2024 Feb 20;25:80. doi: 10.1186/s12859-024-05691-z (PMC10877884; doi:10.1186/s12859-024-05691-z)
Supplement: Supplementary file 1 — Additional file 1. Supplementary tables, figures and pipeline configuration files. [file 12859_2024_5691_MOESM1_ESM.pdf]

Supplementary Material

CytoPipeline & CytoPipelineGUI: A Bioconductor R package suite for building and visualizing automated pre-processing pipelines for flow cytometry data

Philippe Hauchamps[1], Babak Bayat[2], Simon Delandre[2], Mehdi Hamrouni[2], Marie Toussaint[2], Stephane Temmerman[2], Dan Lin[2], Laurent Gatto[1*]

[1*]Computational Biology and Bioinformatics, de duve Institute , UCLouvain, Brussels, Belgium.
[2]GSK, Rixensart,Belgium.

*Corresponding author(s). E-mail(s): laurent.gatto@uclouvain.be;
Contributing authors: philippe.hauchamps@uclouvain.be; babak.bayat@gsk.com; simon.x.delandre@gsk.com; mehdi.hamrouni@gsk.com; marie.x.toussaint@gsk.com; stephane.t.temmerman@gsk.com; dan.8.lin@gsk.com;

Supplementary Tables

Step	Name	*R* package	Version	Method
1	flowframe_read	*flowCore*	2.12.0	*read.flowSet()*
2	remove_margins	*PeacoQC*	1.10.0	*removeMargins())*
3	compensate	*flowCore*	2.12.0	*compensate())*
4	flowframe_aggregate	*CytoPipeline*	1.0.0	*aggregateAndSample())*
5	scale_transform_estimate	*flowCore*	2.12.0	*estimateLogicle())*

Table S1 Scale transformation steps, common to both *PeacoQC*-based and *flowAI*-based pipelines.

Step	Name	*R* package	Version	Method
1	flowframe_read	*flowCore*	2.12.0	*read.FCS()*
2	remove_margins	*PeacoQC*	1.10.0	*removeMargins())*
3	compensate	*flowCore*	2.12.0	*compensate())*
4	perform_QC	*PeacoQC*	1.10.0	*PeacoQC())*
5	remove_doublets	*CytoPipeline*	1.0.0	*removeDoubletsCytoPipeline())*
6	remove_debris	*flowClust*	3.38.0	*tmixFilter())*
7	remove_dead_cells	*flowDensity*	1.34.0	*deGate())*

Table S2 *PeacoQC*-based pipeline pre-processing steps.

Step	Name	*R* package	Version	Method
1	flowframe_read	*flowCore*	2.12.0	*read.FCS()*
2	remove_margins	*PeacoQC*	1.10.0	*removeMargins())*
3	perform_QC	*flowAI*	1.30.0	*flow_auto_qc())*
4	compensate	*flowCore*	2.12.0	*compensate())*
5	remove_doublets	*CytoPipeline*	1.0.0	*removeDoubletsCytoPipeline())*
6	remove_debris	*flowClust*	3.38.0	*tmixFilter())*
7	remove_dead_cells	*flowDensity*	1.34.0	*deGate())*

Table S3 *flowAI*-based pipeline pre-processing steps.

Supplementary Figures

```r
library("CytoPipeline")      # main package
library("CytoPipelineUtils") # adds wrappers to external packages methods

rawDataDir <- "/rawData" # input raw data directory here
jsonDir <- "/pipeDefs"   # input pipeline definition directory here

QCMethod <- "PeacoQC"    # choose which pipeline to run
#QCMethod <- "flowAI"

expName <- paste0("HBVT_ExVivo_", QCMethod)
jsonFile <- file.path(jsonDir,
                      paste0("HBVT_ExVivo_", QCMethod, ".json"))

# locates the 55 raw fcs files
sampleFiles <- list.files(path = rawDataDir, pattern = "*.fcs")

# creates CytoPipeline object
pipL <- CytoPipeline(jsonFile,
                     experimentName = expName,
                     sampleFiles = sampleFiles)

# executes pipeline
execute(pipL)

# monitors the state of the processing queues after the run
plotCytoPipelineProcessingQueue(
    pipL,
    whichQueue = "scale transform")

plotCytoPipelineProcessingQueue(
    pipL,
    whichQueue = "pre-processing",
    sampleFile = 33) # input specific sample file of which to check the run

# interactive visualization using shiny app
library("CytoPipelineGUI")
CytoPipelineGUI::CytoPipelineCheckApp()
```

Fig. S1 Simple sample *R* code to define a *CytoPipeline* object, run it and visualize the results.

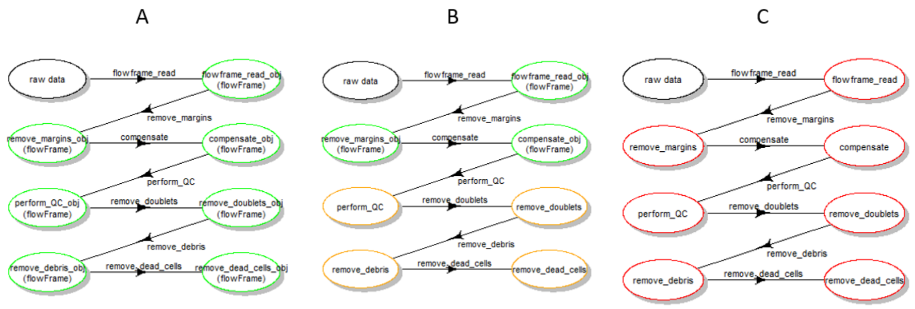

Fig. S2 Illustration of the colour code used to represent the pipeline workflows. Green steps are the ones that have already been run and produced results, indicating a non problematic run. Orange steps are the ones that are correctly defined, but have not run yet for the displayed sample file. Red steps show an inconsitency problem between the already stored results, and the *CytoPipeline* object, meaning that the pipeline was previously run, either with a different number of steps, or with one or several steps that used a different method or different set of parameters. On the above picture, part A shows a situation where part of the pipeline has fully run. Part B shows a situation of pipeline that has run, but not all steps. Part C shows an inconsistency between the pipeline definition and the results stored.

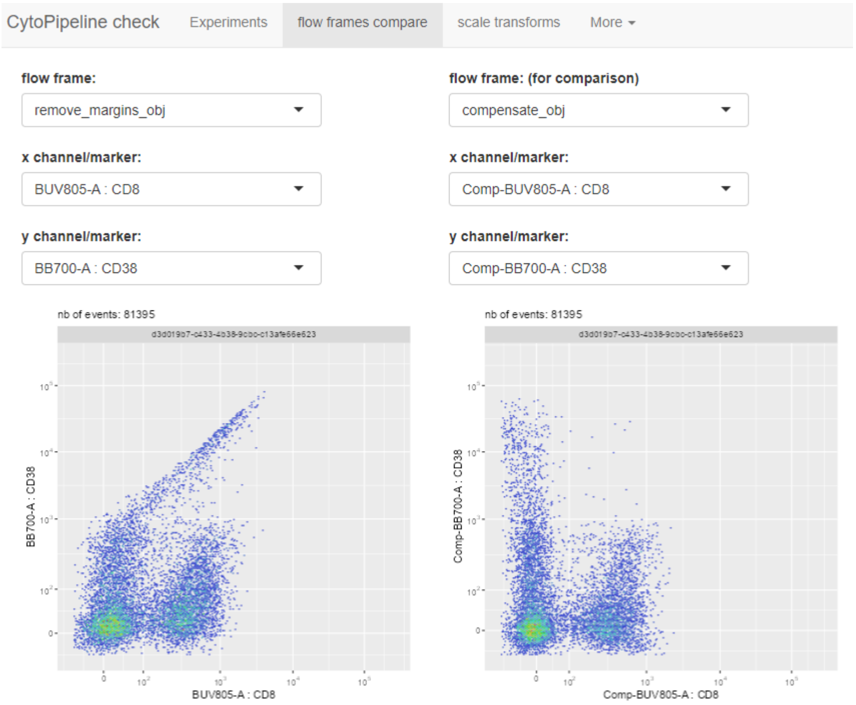

Fig. S3 Screenshot of the interactive GUI application allowing to compare flow frames, and implemented in the *CytoPipelineGUI* package. Here the displayed plots correspond to Figure 4 of the main text.

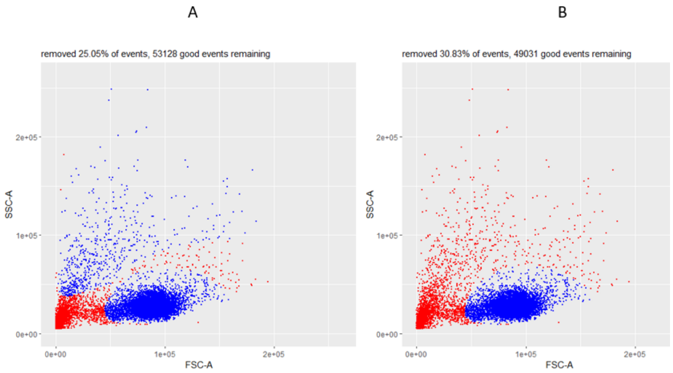

Fig. S4 Comparison between the outcome of debris removal step between the *PeacoQC*-based pipeline with 3 clusters (A), and the *PeacoQC*-based pipeline with 2 clusters (B). The latter setting better eliminates undesirable events than the former.

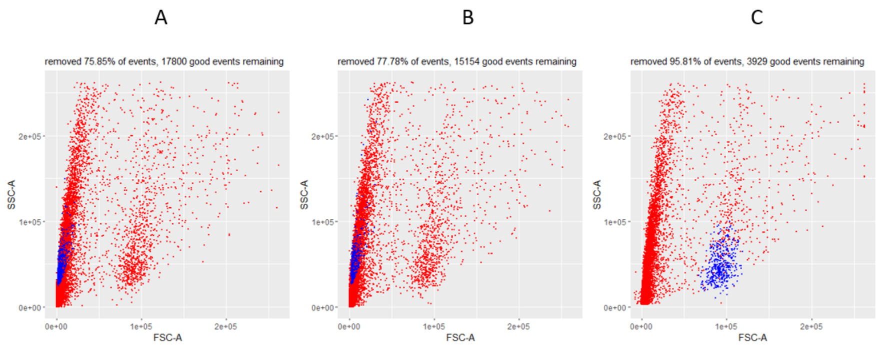

Fig. S5 Comparison between the outcome of three combined steps (doublets, debris and dead cells removals) between the *PeacoQC*-based pipeline (A), the *flowAI*-based pipeline (B), and the ground truth (C), on sample *D93_A05*. Due to the bad quality of this sample, i.e. the majority of the events were composed of debris and dead cells, both automatic pipelines were unable to select the right population of lymphocytes.

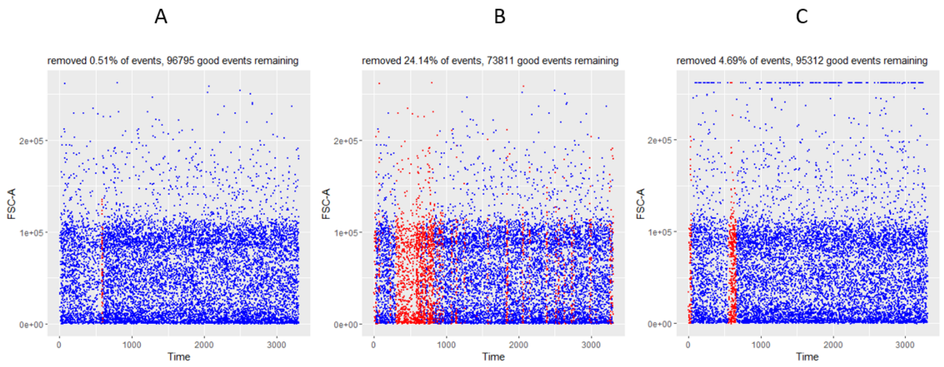

Fig. S6 Comparison between the *QC in time* step with *PeacoQC* (A), with *flowAI* (B), and the time gate applied in manual gating (ground truth) (C), on sample *D91_D03*. *flowAI* removes more event than the ground truth, while *PeacoQC* removes less events.

Supplementary text file 1: json file describing the *PeacoQC*-based pre-processing pipeline


```
{
  "flowFramesPreProcessingSteps": [
    {
      "name": ["flowframe_read"],
      "FUN": ["readSampleFiles"],
      "ARGS": {
        "truncate_max_range": [false],
        "min.limit": null
      }
    },
    {
      "name": ["remove_margins"],
      "FUN": ["removeMarginsPeacoQC"],
      "ARGS": {
        "channelSpecifications": {
          "AllFluoChannels": [-300, 262144]
        }
      }
    },
    {
      "name": ["compensate"],
      "FUN": ["compensateFromMatrix"],
      "ARGS": {
        "matrixSource": ["pData"],
        "pDataVar" : ["day"],
        "pDataPathMapping" : {
            "D91" : ["./compensation/Compensations Liver D91.csv"],
            "D93" : ["./compensation/Compensations Liver D93.csv"]
        }
      }
    },
    {
      "name": ["perform_QC"],
      "FUN": ["qualityControlPeacoQC"],
      "ARGS": {
        "preTransform": [true],
        "min_cells": [150],
        "max_bins": [500],
        "step": [500],
        "MAD": [6],
        "IT_limit": [0.6],
        "force_IT": [150],
        "peak_removal": [0.3333],
        "min_nr_bins_peakdetection": [10]
      }
    },
    {
      "name": ["remove_doublets"],
      "FUN": ["removeDoubletsCytoPipeline"],
      "ARGS": {
        "areaChannels": ["FSC-A"],
        "heightChannels": ["FSC-H"],
        "nmads": [3]
      }
    },
    {
      "name": ["remove_debris"],
      "FUN": ["removeDebrisFlowClustTmix"],
      "ARGS": {
        "FSCChannel": ["FSC-A"],
        "SSCChannel": ["SSC-A"],
        "nClust": [2],
        "level": [0.97],
        "B": [100],
        "verbose": [true]
      }
    },
    {
      "name": ["remove_dead_cells"],
      "FUN": ["removeDeadCellsDeGate"],
      "ARGS": {
                "preTransform": [true],
        "LDMarker": ["Live \& Dead"]
      }
    }
  \],
   "scaleTransformProcessingSteps": [
    {
      "name": ["flowframe_read"],
      "FUN": ["readSampleFiles"],
      "ARGS": {
        "whichSamples": ["random"],
        "nSamples": [4],
```

```
        "seed": [0],
        "truncate_max_range": [false],
        "min.limit": null
      }
    },
    {
      "name": ["remove_margins"],
      "FUN": ["removeMarginsPeacoQC"],
      "ARGS": {
        "channelSpecifications": {
          "AllFluoChannels": [-300, 262144]
        }
      }
    },
    {
      "name": ["compensate"],
      "FUN": ["compensateFromMatrix"],
      "ARGS": {
        "matrixSource": ["pData"],
        "pDataVar" : ["day"],
        "pDataPathMapping" : {
            "D91" : ["./compensation/Compensations Liver D91.csv"],
            "D93" : ["./compensation/Compensations Liver D93.csv"]
        }
      }
    },
    {
      "name": ["flowframe_aggregate"],
      "FUN": ["aggregateAndSample"],
      "ARGS": {
        "nTotalEvents": [10000],
        "seed": [0]
      }
    },
    {
      "name": ["scale_transform_estimate"],
      "FUN": ["estimateScaleTransforms"],
      "ARGS": {
        "fluoMethod": ["estimateLogicle"],
        "scatterMethod": ["linearQuantile"],
        "scatterRefMarker": ["CD4"]
      }
    }
  ]
}
```

Supplementary text file 2: json file describing the *flowAI*-based pre-processing pipeline

```
{
  "flowFramesPreProcessingSteps": [
    {
      "name": ["flowframe_read"],
      "FUN": ["readSampleFiles"],
      "ARGS": {
        "truncate_max_range": [false],
        "min.limit": null
      }
    },
    {
      "name": ["remove_margins"],
      "FUN": ["removeMarginsPeacoQC"],
      "ARGS": {
        "channelSpecifications": {
          "AllFluoChannels": [-300, 262144]
        }
      }
    },
    {
      "name": ["perform_QC"],
      "FUN": ["qualityControlFlowAI"],
      "ARGS": {
        "preTransform": [false],
        "remove_from": ["all"],
        "second_fractionFR": [0.1],
        "deviationFR": ["MAD"],
        "alphaFR": [0.01],
        "decompFR": [true],
        "outlier_binsFS": [false],
        "pen_valueFS": [500],
        "max_cptFS": [3],
                "sideFM": ["both"],
                "neg_valuesFM": [1]
      }
    },
    {
      "name": ["compensate"],
      "FUN": ["compensateFromMatrix"],
      "ARGS": {
        "matrixSource": ["pData"],
        "pDataVar" : ["day"],
        "pDataPathMapping" : {
            "D91" : ["./compensation/Compensations Liver D91.csv"],
            "D93" : ["./compensation/Compensations Liver D93.csv"]
        }
      }
    },
    {
      "name": ["remove_doublets"],
      "FUN": ["removeDoubletsCytoPipeline"],
      "ARGS": {
        "areaChannels": ["FSC-A"],
        "heightChannels": ["FSC-H"],
        "nmads": [3]
      }
    },
    {
      "name": ["remove_debris"],
      "FUN": ["removeDebrisFlowClustTmix"],
      "ARGS": {
        "FSCChannel": ["FSC-A"],
        "SSCChannel": ["SSC-A"],
        "nClust": [2],
        "level": [0.97],
        "B": [100],
        "verbose": [true]
      }
    },
    {
      "name": ["remove_dead_cells"],
      "FUN": ["removeDeadCellsDeGate"],
      "ARGS": {
                "preTransform": [true],
        "LDMarker": ["Live \& Dead"]
      }
    }
  ],
  "scaleTransformProcessingSteps": [
    {
      "name": ["flowframe_read"],
      "FUN": ["readSampleFiles"],
      "ARGS": {
```

```
        "whichSamples": ["random"],
        "nSamples": [4],
        "seed": [0],
        "truncate_max_range": [false],
        "min.limit": null
      }
    },
    {
      "name": ["remove_margins"],
      "FUN": ["removeMarginsPeacoQC"],
      "ARGS": {
        "channelSpecifications": {
          "AllFluoChannels": [-300, 262144]
        }
      }
    },
    {
      "name": ["compensate"],
      "FUN": ["compensateFromMatrix"],
      "ARGS": {
        "matrixSource": ["pData"],
        "pDataVar" : ["day"],
        "pDataPathMapping" : {
            "D91" : ["./compensation/Compensations Liver D91.csv"],
            "D93" : ["./compensation/Compensations Liver D93.csv"]
        }
      }
    },
    {
      "name": ["flowframe_aggregate"],
      "FUN": ["aggregateAndSample"],
      "ARGS": {
        "nTotalEvents": [10000],
        "seed": [0]
      }
    },
    {
      "name": ["scale_transform_estimate"],
      "FUN": ["estimateScaleTransforms"],
      "ARGS": {
        "fluoMethod": ["estimateLogicle"],
        "scatterMethod": ["linearQuantile"],
        "scatterRefMarker": ["CD4"]
      }
    }
  ]
}
```
